# Maintenance of Genome Integrity: How Mammalian Cells Orchestrate Genome Duplication by Coordinating Replicative and Specialized DNA Polymerases

**DOI:** 10.3390/genes8010019

**Published:** 2017-01-06

**Authors:** Ryan Barnes, Kristin Eckert

**Affiliations:** 1 Biomedical Sciences Graduate Program, Pennsylvania State University College of Medicine, Hershey, PA 17033, USA; rbarnes1@hmc.psu.edu; 2 Departments of Pathology and Biochemistry & Molecular Biology, The Jake Gittlen Laboratories for Cancer Research, Pennsylvania State University College of Medicine, Hershey, PA 17033, USA

**Keywords:** translesion synthesis, replication stress, transcriptional regulation, polymerase interactions, polymerase domains, polymerase modifications

## Abstract

Precise duplication of the human genome is challenging due to both its size and sequence complexity. DNA polymerase errors made during replication, repair or recombination are central to creating mutations that drive cancer and aging. Here, we address the regulation of human DNA polymerases, specifically how human cells orchestrate DNA polymerases in the face of stress to complete replication and maintain genome stability. DNA polymerases of the B-family are uniquely adept at accurate genome replication, but there are numerous situations in which one or more additional DNA polymerases are required to complete genome replication. Polymerases of the Y-family have been extensively studied in the bypass of DNA lesions; however, recent research has revealed that these polymerases play important roles in normal human physiology. Replication stress is widely cited as contributing to genome instability, and is caused by conditions leading to slowed or stalled DNA replication. Common Fragile Sites epitomize “difficult to replicate” genome regions that are particularly vulnerable to replication stress, and are associated with DNA breakage and structural variation. In this review, we summarize the roles of both the replicative and Y-family polymerases in human cells, and focus on how these activities are regulated during normal and perturbed genome replication.

## 1. Introduction

Human cells encode 15 distinct nuclear DNA polymerases with widely varying enzymatic properties and accuracies, reflecting the need for biochemical flexibility during genome maintenance. DNA polymerases of the Y-family are characterized by their unique ability to efficiently replicate non-B DNA structures, as well as numerous DNA lesions formed by endogenous cellular processes and exposure to exogenous agents (reviewed in [1,2]). The need for this enzymatic flexibility during DNA replication is reflected in the conservation of the Y-family from *E. coli* and yeast, to rodents and mammals. DNA polymerase errors during DNA synthesis pathways associated with replication, repair, and recombination can cause mutations that drive cancer and aging. Y-family polymerases, although essential, have higher error rates than replicative polymerases. While the biochemistry of DNA lesion bypass or translesion synthesis (TLS) by Y-family polymerases has been extensively studied (reviewed in [3]), the regulation of these polymerases is often viewed in that narrow context, and how mammalian cells orchestrate DNA polymerase activities to maintain genome stability is an open question. In this review, we summarize the factors regulating both the expression and activity of the Y-family polymerases, focusing primarily on mammalian cells, and compare such regulation to the major replicative polymerases of the B-family.

## 2. Overview of Polymerase Functions

Currently known functions of the mammalian DNA polymerases to be discussed in this review, as well as gene and protein nomenclature, are summarized in Table 1. Replication of the human genome is carried out primarily by the replicative B-family polymerases (pols) α, δ, and ε [4]. The coordinated activities of several DNA polymerases are required for DNA repair pathways including base excision repair (BER), nucleotide excision repair (NER), mismatch repair (MMR), double-strand break repair (DSBR), and homologous recombination (HR) (Table 1). In response to replication stress, the ATR-mediated intra-S phase checkpoint coordinates DNA replication, repair and recombination processes at stalled replication forks [5]. Polymerases required to activate the ATR checkpoint include Pol α, Pol κ, Pol δ and Pol ε (Table 1). The replisome is a highly dynamic structure, and current models to explain resolution of stalled replication forks specialized polymerases (Y-family and Pol ζ) include performing DNA synthesis at the fork when replicative polymerases (B-family) are inhibited, or post-replicative gap-filling synthesis behind the replication fork [6]. Repetitive sequences make up ~67% of the human genome [7], and are enriched within rare and common fragile sites (CFS), chromosomal regions susceptible to breakage, particularly under replication stress [8]. Our laboratory has proposed that the presence of multiple DNA polymerases with complementary biochemical activities and accuracies reflects the complexity of completing DNA replication in genomes with a high density of repetitive DNA sequences [9]. We demonstrated biochemically that microsatellite sequences and high flexibility AT-rich repeats are particularly inhibitory to replicative DNA α and δ polymerase elongation [9,10,11]. We also made the novel discovery that Pols η and κ efficiently replicate through repetitive DNA sequences [11,12]. Loss of either Pol η or Pol ξ increases CFS breakage [13,14], underscoring the importance of these enzymes in maintaining genome integrity. While classified as a Y-family polymerase gene, REV1’s catalytic terminal transferase activity is overwhelmingly dispensable [15]. Instead, REV1’s crucial function is to serve as a scaffolding protein and assist the function of other polymerases.

Although they have dramatically different biochemical capacities (i.e., DNA synthesis efficiency and fidelity using defined DNA substrates), the B- and Y-family DNA polymerases adopt the conserved “right hand” structure found in polymerases of all forms of life. B-family polymerases utilize the thumb domain to make extensive contact with both the primer and template DNA; the palm domain contains catalytic residues and coordinates the Mg^2+^ ions; and the finger domain makes extensive conformational swings to coordinate the incoming deoxynucleotide (dNTP) with the template. While the Y-family enzymes share these general features and similar overall structure, they have several key differences. Foremost, all of the Y-family polymerases have a little finger domain which compensates for diminished interaction of the thumb domain with the template. Compared to B-family polymerases, the finger domains are also smaller and more rigid, making little-to-no movement when interacting with a dNTP, leaving the active site largely solvent exposed [16]. This finding has led to the proposal that in contrast to the tight complex between polymerase, DNA, and dNTP made by the fingers of B-family polymerases, Y-family polymerases have a preformed active site, explaining their low fidelity and catalytic efficiency [17].

Moreover, the little finger domain is believed to ascribe unique biochemical functions to certain polymerases. For Pol η, this domain was shown to interact tightly with the catalytic core and act as a molecular splint, forcing DNA to adopt a B-form structure in the active site [18]. This ability may explain Pol η’s ability to both accurately replicate certain DNA lesions, as well as repetitive DNA [2,17,19]. Pol κ also contains an N-terminal clasp domain which allows it to encircle DNA, linking the little finger and thumb, while also interacting with the primer [20]. In contrast to Pol η, Pol κ has a large gap between the little finger and thumb domain which may accommodate bulky minor groove lesions [21]. Indeed, recent structural work has shown that the bulky benzo[a]pyrene adduct is easily accommodated by the Pol κ active site, as the adducted DNA remained in B-form, displaying little difference to normal DNA [22]. This finding suggests that the function of specialized polymerases to replicate non-B DNA may be a result of their ability to force DNA into a B-form.

## 3. DNA Polymerase Expression during an Unperturbed Mitotic Cell Cycle

All four Y-family polymerases are expressed throughout the adult organism in mice and humans. Comparatively, the expression of *POLH*, *POLK*, and *REV1* genes is high in testis and ovaries, moderate in tissues such as kidney, liver, and spleen, and low in slow proliferating tissues, such as skeletal muscle and brain [39,40,41,42,43]. The *POLI* gene is expressed highly in testis and ovaries and present in other adult human tissues, but at low levels [44,45]. The Y-family polymerase proteins are expressed at very low levels, with as few as 60,000 molecules of Pol η and REV1 estimated in unperturbed human cells [46]. For comparison, each human cell is estimated to have ~3 million molecules of Pol ε and 500,000 molecules of Pol δ, based on the abundance of the catalytic subunits [47]. Additionally, unlike replicative polymerases and PCNA which increase transcript and protein just before S-phase, Y-family polymerases either do not change expression during the cell cycle (*POLI* and *REV1*) or increase only in G2/M (*POLH*) [48].

### 3.1. Transcriptional Regulation 

#### 3.1.1. Sp1

**Figure 1:**
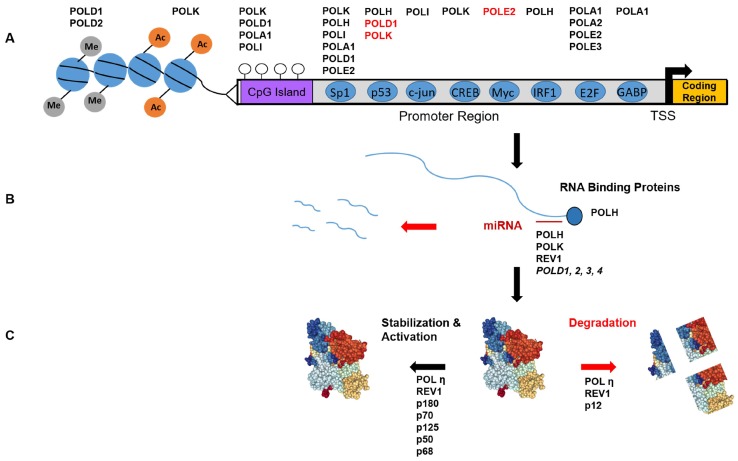
Overview of DNA polymerase regulation. (**A**) Transcriptional regulation of the B and Y-family polymerases genes (Top, see Table 1), as controlled by histone modifications (grey and orange circles), CpG methylation (open circles), and transcription factors (blue circles). Genes in red are negatively regulated by the factor below. TSS = transcription start site; (**B**) Post-transcriptional regulation: Polymerase mRNA stability is controlled by mRNA binding proteins and microRNA binding at the 3′ UTR; (**C**) Post-translational regulation: Polymerase proteins can be stabilized and functionally activated by various modifications, or prompted for degradation (red). See text for details.

The Sp1 transcription factor regulates numerous genes in processes such as apoptosis, cell growth, and the immune response [49]. Sp1 regulation of basal transcription has been functionally characterized for several mammalian replicative polymerase genes (*POLA1*, *POLD1*, *POLE2*) [50,51,52], and both direct and indirect evidence suggests that Sp1 regulation extends to all four Y-family polymerase genes (Figure 1A).

Early work characterizing the *POLK* promoter showed the presence of both *cis* repressive (−1413/−395) and activating elements (−395/−83) [53]. Mutation of a CREB binding element or an Sp1 site (−180 and −78 respectively) reduced *POLK* promoter activity, as measured using luciferase reporter constructs (pGL3-Basic). Indeed, these proteins were shown to bind their cognate sequences in vitro by mobility shift assays, and over-expression of CREB, Sp1, or Sp3 enhanced luciferase expression via the *POLK* promoter. The *POLK* gene also harbors an Sp1 motif at position +60, and this upstream site was confirmed to positively regulate *POLK* [54]. However, several other putative transcription factor binding sites were shown not to affect *POLK* promoter activity, including SMAD and NFκB.

The *POLI* promoter contains functional Sp1 binding motifs [55], and an early study documented the control of *POLI* expression by Sp1 [56]. Using chromatin immunoprecipitation (ChIP), Sp1 was confirmed to bind within the *POLI* promoter, and overexpression of Sp1 enhanced luciferase expression driven by the *POLI* promoter. Interestingly, overexpression of Sp1, but not Oct-1, increased *POLI* mRNA, despite the presence of predicted Oct-1 sites.

The *POLH* gene promoter also contains putative Sp1 motifs, and deletion of Sp1 motifs reduced luciferase expression to levels comparable to the empty pGL3 vector [57]. Additionally, publicly available ChIP data (UCSC Genome Browser, [58]) shows an Sp1 signal in the *POLH* promoter, at the consensus sequence. To our knowledge, there are no reports characterizing the human *REV1* promoter. However, the UCSC Genome Browser also shows an Sp1 ChIP signal at the 3′ of the *REV1* gene in intron 17. Although this peak lacks the consensus motif, a possible binding site can be found in intron 22 (5′-AGGGCGGATC-3′) and several 5′-GGGCGG-3′ motifs are present in the promoter region.

#### 3.1.2. p53

The p53 transcription factor was first reported a decade ago to positively regulate the *POLH* [59]. Overexpression of *TP53* increased Pol η mRNA levels and enhanced luciferase activity in a reporter assay. Importantly, overexpression of a mutant *TP53* (R175H) was unable to enhance luciferase expression. Unpublished data from our laboratory and other studies have confirmed higher expression levels of human *POLH* in *TP53* proficient cells compared to deficient cells [60]. *POLH* gene regulation by p53 is conserved in murine cells [61].

In contrast, the effect of p53 on *POLK* expression appears to have diverged between humans and mice. While murine *POLK* expression is enhanced by p53 in the absence of DNA damage, human *POLK* expression is either unaffected [39], or negatively regulated [62]. In the latter study, luciferase constructs containing the human *POLK* promoter were inhibited when *TP53* was transiently overexpressed, compared to controls, and this inhibition was dependent on p53 DNA binding activity [62]. Consistently, using the same human constructs in mouse cells, *POLK* promoter-dependent luciferase activity was increased in *TP53* null cells, compared to wild-type. Similar to *POLK*, *POLD1* gene transcription is repressed by p53 binding to the core promoter, in a mechanism that excludes Sp1 binding [63,64].

#### 3.1.3. E2F

Replicative polymerase gene transcription is increased upon mitogen-stimulated entry into the mitotic cell cycle after serum deprivation (G0) growth arrest. This response has been functionally characterized for several genes (*POLA1, POLA2, POLD1, POLE2*, and *POLE3)*, and is dependent upon E2F transcription factor binding [50,51,52,65,66].

#### 3.1.4. Epigenetic Regulation

Very little is known regarding the epigenetic regulation of polymerase genes. The promoters of both *POLI* and *POLK* are unmethylated and treatment of cells with 5-azacytidine did not alter expression [53,55]. In these same studies, treatment with Trichostatin A and did not change the expression of *POLI*, but *POLK* expression was increased ~five-fold, suggesting histone acetylation status is used to regulate *POLK* expression. *POLA1* and *POLD1* gene expression is also unresponsive to 5-azacytidine and Trichostatin A suggesting an absence of repressive epigenetic modification at their promoters [53]. However, the PRMT7 histone methyltransferase is a negative regulator for *POLD1* and *POLD2* gene expression [67].

### 3.2. Post-Transcriptional Regulation

An important point for regulating gene expression is at the level of the mRNA half-life. The stability of *POLH* mRNA is enhanced by binding of PRCB1 (or hnRNP E1) to an AU-rich element within the 3′ UTR [68] (Figure 1B). Knock-down of this protein reduces Pol η protein levels via a reduction in *POLH* mRNA half-life.

Overexpression of miR-155 causes down-regulation of all four *POLD* genes [69], but it is unclear whether miR-155 regulates Pol δ expression by directly binding to *POLD* gene transcripts. However, micoRNAs have been shown to regulate most of the Y-Family polymerases. miR-96 negatively regulates *REV1* in human cells by interacting with a predicated binding site in the 3′ UTR [70]. miR-20b is predicated to bind the 3′ UTR of both *POLH* and *POLK* transcripts, and the miR-20b binding site was confirmed to be functional for the *POLK* 3′UTR. Overexpressing a miR-20b mimic reduces, while a miR-20b inhibitor elevates, Pol κ protein levels [71]. In a separate report, the downregulation of miR-93 expression in ovarian cancer cells caused an increase in Pol η levels. This negative regulation was validated using both a miR-93 mimic and an inhibitor [72]. In contrast to the study by Guo et al. [72], *POLK* transcript was not affected even though miR-20b was downregulated in the ovarian cancer cells, and a miR-20b mimic did not alter Pol η expression [72].

### 3.3. Post-Translational Modifications-Functional 

Phosphorylation is an important mechanism regulating replicative polymerases (Figure 1C). Pol α-primase holoenzyme activity is regulated by cyclin-dependent kinases (CDKs) in a cell cycle-dependent manner [73]. The p180 catalytic subunit is a phosphoprotein that becomes hyperphosphorylated in G_2_/M phase, while the regulatory p70 subunit is phosphorylated only in G_2_/M [74]. Pol α phosphorylation results in lowered single-stranded DNA binding affinity, lowered DNA synthesis activity, and an inhibition of DNA replication [73,74].

The mammalian Pol δ holoenzyme consists of catalytic p125 (POLD1), regulatory p50 (POLD2), regulatory p68 (POLD3) and p12 (POLD4) subunits [75]. The Pol δ holoenzyme is phosphorylated in a cell cycle-dependent manner (see [76] for review). The catalytic p125 subunit is phosphorylated primarily during S-phase [77]. The regulatory B subunit (p50) is phosphorylated in vivo, and is an in vitro substrate of the Cyclin A-CDK2 cell cycle-dependent kinase [78]. The regulatory C subunit (p68) can be phosphorylated by G1/S phase and S-phase cyclin-dependent kinases in vitro, and PCNA interferes with this phosphorylation [79]. Phosphorylation of p68 coincides with Pol δ association with chromatin at the start of S-phase [80]. The regulatory p68 subunit also contains a phosphorylation site for Protein Kinase A, and phosphomimetic mutation of this residue decreases Pol δ affinity for PCNA and processivity [81]. In addition, mammalian p125, p68 and p12 subunits can be phosphorylated by Casein Kinase 2 in vitro, and subsequently dephosphorylated by protein phosphatase-1 [82], suggesting an additional regulatory circuit for regulation. Thus, phosphorylation may serve to regulate Pol δ activity by controlling its interaction with DNA and/or auxiliary proteins during replication.

### 3.4. Post-Translational Modifications-Degradation

While the mammalian Pol δ holoenzyme is a heterotetrameric protein (Pol δ4), the Pol δ holoenzyme found in budding yeast, *Saccharomyces cerevisiae*, lacks the small subunit, and exists only in the three subunits assembly (Pol δ3). The human p12 subunit interacts with the p125 and p50 subunits, increasing stability of the Pol δ holoenzyme and increasing PCNA-dependent DNA synthesis activity [78]. During an unperturbed mitotic cell cycle, p12 levels fall during G1 phase, preceding the initiation of DNA synthesis, and rapidly rise again upon completion of DNA synthesis and transition to the G2/M phase [83] (Figure 1C). During S- phase, the majority of Pol δ activity is attributed to the Pol δ3 form [83,84]. This partial degradation of p12 occurs via a PCNA interacting peptide (PIP) degron sequence and is controlled by the CRL4^Cdt2^ E3 ligase [85]. CRL4^Cdt2^ recognizes substrates bound to chromatin-loaded PCNA and is a key regulator of replication [86]. Another CRL4^Cdt2^ substrate, the p21 protein, directly interacts with the PolD2/p50 subunit, and p21 and p12 are coordinately degraded in S-phase. The biochemical properties of the human Pol δ3 and Pol δ4 forms differ, with the Pol δ3 form being more adapted for completion of Okazaki fragment processing and DNA repair synthesis [87].

## 4. DNA Polymerase Expression under Stress

### 4.1. Transcriptional Regulation

The cellular response to ultraviolet (UV) radiation in human cells involves upregulation of Pol ι expression [88] that is dependent on ATR activation of c-jun [89] (Figure 1A). This is surprising, considering Pol ι over-expression is unable to rescue the UV sensitivity of patient derived, Pol η-deficient cells [90]. In mice, UV induces Pol κ expression in a p53-dependent manner, whereas in human cells, UV induces either no change (p53 positive cells) or a reduction (p53 deficient cells) in *POLK* expression [39]. Notably, *POLK* was upregulated following UV in patient derived Pol η-deficient cells [91], suggesting a regulatory adaptation to loss of Pol η. Surprisingly, despite its function as an accurate and efficient TLS polymerase for UV induced pyrimidine dimers, the levels of Pol η actually decrease following UV irradiation in human and murine cells [42,92] (see Section 4.3).

Pol η levels are induced following treatments that create double strand breaks. Exposure to both camptothecin (CPT) or ionizing radiation (IR) induces transactivation of *POLH* in human cells in a p53-dependent manner [59]. Studies examining the relationship between p53 and Pol κ have produced interesting results. Murine cells treated with doxorubicin, which can cause strand breaks, causes *POLK* upregulation in a p53 dependent manner, whereas similar treatment of human cells caused either a reduction (p53 deficient) or no change (p53 wild-type) in *POLK* expression [39]. These findings are consistent with the basal levels of *POLK* mentioned above, and provide further support that p53’s role in Y-family polymerase regulation has diverged between rodents and primates.

Notably, *POLH*, *POLI*, and *POLK* gene expression are all induced by alkylation damage. *N*-methyl-*N*’-nitro-*N*-nitrosoguanidine treatment of human cells induces *POLH* expression in a pathway dependent on interferon regulatory factor 1 (IRF1), and *POLI* gene expression in an Sp1 dependent manner [56,57]. Temozolomide, an alkylating drug used in chemotherapy, upregulates the expression of Pol κ at both the mRNA and protein levels [93].

### 4.2. Post-Translational Modifications—Functional

#### 4.2.1. Phosphorylation

Early work showed that following UV radiation, human Pol η was phosphorylated and its foci formation was reduced in ATR-depleted cells [94]. Later work demonstrated that Pol η is directly phosphorylated at Ser 601 by ATR in vitro and in cells following UV, hydroxyurea, cisplatin, and CPT treatment (Figure 1C). This phosphorylation is dependent on Pol η‘s interaction with Rad18 and Pol η‘s ubiquitin binding zinc finger (UBZ) domain but independent of Rad18 catalytic activity and PCNA ubiquitination [95]. Importantly, following UV treatment, loss of Ser601 phosphorylation does not impact Pol η chromatin localization or foci formation, but does reduce cell survival. A recent report using LC-MS/MS discovered that both Ser601 and Ser687 are phosphorylated in untreated human cells [96]. The Ser687 phosphorylation is induced following UV by Cyclin A2- CDK2 [96]. Again, loss of this Ser687 phosphorylation does not impact Pol η nuclear localization but does reduce cell viability following UV treatment. Interestingly, a phospho-mimetic mutant (S687D) had reduced PCNA interaction, but no defect in a cellular TLS assay compared to wild-type cells. These findings together suggest that despite reduced PCNA interaction, Pol η phosphorylation promotes its activity. Further biochemical studies are required to determine if phosphorylation impacts Pol η activity per se.

#### 4.2.2. Ubiquitination

Multiple reports have shown that Pol η is mono-ubiquitinated at Lys682, under normal conditions [96,97]. The nuclear localization sequence of Pol η, including Lys682, Lys686, Lys694, and Lys709, is ubiquitinated in cells by the PIRH2 E3 ligase [98], and these modifications act as a surface for PCNA interaction along with the PIP box. In response to UV, ubiquitinated Pol η disappears, suggesting that removal of the modification is required for function [97]. Consistent with this, a ubiquitin-Pol η chimera has reduced nuclear foci and compromised PCNA interaction, due to intramolecular interaction between ubiquitin and the UBZ domain of Pol η [97,98]. However, this mutant has only slightly reduced clonogenic survival in comparison to wild-type cells [97,98]. These studies suggest that while mono-ubiquitination is a bona-fide mechanism for regulating Pol η/PCNA interaction and foci formation, this modification does not dramatically reduce its ability to prevent UV sensitivity, especially in comparison to Pol η-deficient cells.

In contrast to Pol η, there is little experimental evidence concerning functional consequences of post-translational modifications of the other Y-family polymerases, although all three are ubiquitinated in a UBZ/UBD dependent fashion [99,100,101,102,103]. The E3 ligase TRIP has been shown to interact with Pol κ [104]. Polymerase δ subunits p68 and p12 are modified by ubiquitination, and the p68 subunit is primarily mono-ubiquitinated [105]. The p68 subunit also is SUMOylated by SUMO3 in unperturbed cells [105] and by SUMO2 in cells under replication stress [106]. The functional consequences of the p68 SUMOylation have not been determined, although the lysine residues modified by SUMO3 lie outside of the known p50 and PCNA binding domains.

### 4.3. Post-Translational Modifications—Degradation and Stability

Human Pol η is degraded following UV treatment by the proteasome. MDM2 negatively regulates Pol η following UV via poly-ubiquitination [107], while, USP7 acts as a de-ubiquitinase, preventing the poly-ubiquitination of Pol η and its degradation [108]. USP7 also negatively regulates the stability of MDM2. Thus, MDM2 and USP7 regulate Pol η levels in an inverse manner. Following UV treatment, Pol η is also targeted for degradation in a ubiquitin independent fashion by PIRH2 [92]. TRIP is also known to poly-ubiquitinate human Pol η, but the consequences of this on stability have not been examined [104]. In *C. elegans*, degradation of Pol η is prevented by GEI-17 [109]. GEI-17 (PIAS homolog) SUMOylates Pol η to halt CRL4-Cdt2 mediated degradation following UV and methyl methanesulfonate (MMS). An epistatic relationship between *C. elegans* Pol η and GEI-17 following UV confirms that the two proteins act in the same pathway [110]. Recently, the SUMOylation of human Pol η was shown to occur following UV and replication stress, although its functional consequence is unclear [111]. PIAS1 acts as the SUMO ligase for human Pol η and its function depends on Rad18. *POLK* was also implicated to act in the GEI-17 pathway in the *C. elegans* study, and identified as a SUMOylated peptide in a human cell proteomic screen [112], but there have been no reports validating human Pol κ SUMOylation. Finally, REV1 is SUMOylated by PIAS in a cell model of doxorubicin sensitization by starvation [113]. This modification promotes REV1 stability, and was also demonstrated in H_2_O_2_ treated cells. Since PIAS is the human homolog of *C. elegans* GEI-17, the data to date suggest that SUMOylation is a conserved mechanism for regulating Y-family polymerases.

Degradation of the human Pol δ p12 subunit (Section II D) also has been studied during the DNA damage response (reviewed in [76]). Upon treatment of cells with various DNA damage-inducing agents, including UV, MMS, hydroxyurea, aphidicolin, and IR, the p12 subunit undergoes complete ubiquitylation-dependent degradation, to form Pol δ3. This process requires both the CRL4^Cdt2^ and the RNF8 E3 ligases [84,114]. Under conditions of low UV doses, Pol δ3 formation is dependent on activation of ATR [115]. After UV irradiation, ~70% of cells with cyclobutane pyrimidine dimer foci co-localize with the Pol δ3 form exclusively, consistent with a role for Pol δ3 in NER re-synthesis [116]. Interestingly, Pol δ3 displays increased exonuclease partitioning and decreased potential for bypass of various DNA lesions [76]. These findings led to a model in which Pol δ3 may slow fork replication progression at sites of DNA damage, allowing for switching to a specialized DNA polymerase [76].

Both Pol η and REV1 interact with Hsp90, suggesting that proper folding of polymerases is required for optimal function [117,118]. Inhibition of this interaction reduced UV induced foci formation of both polymerases and, in some cell lines, reduced protein stability. Inhibition of Hsp90 also reduced the interaction of these polymerases with PCNA after UV and was epistatic to knock-down of either protein in UV induced mutagenesis.

## 5. Orchestration of DNA Polymerases

**Figure 2:**
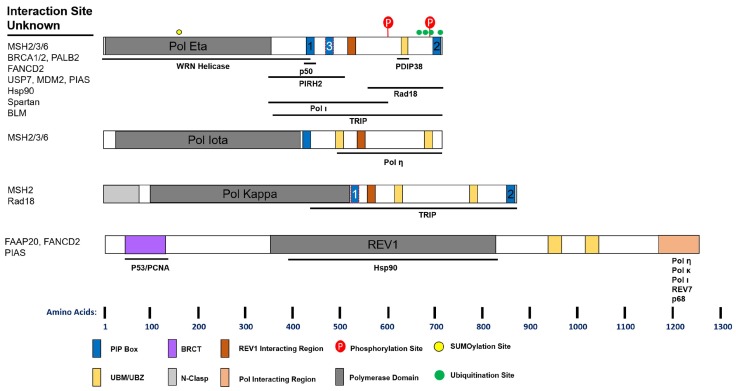
Schematic of Y-Family Polymerase Domains and Interaction Sites. Functional domains that have been experimentally validated are indicated and drawn to scale along the length of the protein. PIP (PCNA Interacting Peptide) boxes with red highlight are putative. Below each cartoon are the known sites of interaction between the polymerase and the indicated protein. Proteins whose interaction has been suggested but the precise site is unknown are listed to the left. See text for details.

Maintenance of genome stability requires the formation of distinct replication and repair complexes that include both replicative and specialized DNA polymerases. A summary of known protein interactions of the Y-family polymerases is given in Figure 2. The regulation of Y-family polymerase complex formation is fairly well studied, and cross-talk between the B- and Y-family polymerases also occurs through several mechanisms. Human Pol η directly interacts with the p50 subunit of Pol δ, but Pol κ and ι do not [119]. This interaction occurs via an FF motif (F1) and is required for optimal UV survival, but not Pol η foci formation. Mutation of this site also reduced the cellular interaction between Pol η and p50, as well as Pol η and PCNA. The F1 motif was later described as PIP3, and while still putative, may explain the latter phenotype [120].

Human REV1 harbors a C-terminal domain capable of interacting with the other Y-family polymerases, REV7, and the p68 subunit of Pol δ [121] (Figure 2). Interaction with REV1 promotes Pols η, κ, and ι UV-induced foci formation and UV lesion TLS [122]. Structural studies suggest that REV1 may orchestrate multiple polymerases simultaneously [123,124]. This is of particular interest as REV1 interacts with p68 in a complex similar to Pol η or κ [125]. Moreover, this may be a function unique to higher organisms, as binding of REV1 to Y-family polymerases is found in humans, mice, and flies, but not worms and yeast [126]. Further studies are required to assess the cellular consequences of these interactions.

PDIP38 also provides a link between Pol δ and specialized polymerases, binding both p50 and Pol η [127,128]. This protein interacts with Pol η in an area overlapping the UBZ, but independent of ubiquitin binding. Loss of PDIP38 does not reduce Pol η foci following UV, but does impair viability to the same extent as Pol η knock-down. Moreover, PDIP38 interacts with REV1, but not Pols κ and ι. Combined with the above report, it seems PDIP38 may facilitate the interaction between Pol δ and η and thereby promote TLS following UV. Several questions remain, however, as to the biochemical consequences of these interactions.

Spartan (C1orf124), a ubiquitin binding protein, was recently identified as a regulator of TLS. Spartan interacts directly with the p68 subunit of Pol δ through its zinc metalloprotease domain [129]. Spartan interaction with p68 is lost following UV, and Spartan interacts strongly with Pol η instead. Depletion of Spartan increases the interaction of p68 with REV1, and reduces UV induced Pol η foci, suggesting that Spartan positively regulates TLS [130,131]. Although controversial, Spartan is proposed to positively regulate TLS by promoting Rad18 activity, PCNA ubiquitination, and inhibiting USP1 [130,132,133]. However, it has also been inferred from mutagenesis studies that Spartan negatively regulates TLS [129,133,134]. In these reports, knock-down of Spartan elevates UV mutagenesis. However, these studies were conducted in *POLH* and NER proficient cells, which actually suggests that Spartan either suppresses erroneous repair processes, or promotes error-free pathways. In agreement with this, knock-down of REV1, which promotes error-prone TLS, eliminates the increased mutagenesis in Spartan depleted cells [129]. Finally, Spartan depletion sensitizes cells to UV, again suggesting that it promotes TLS activity [130,131]. Future studies examining the epistasis between Spartan and accurate TLS mechanisms, as well as the functional consequences of Spartan interactions, are required.

There is growing evidence that the Fanconi anemia (FA) pathway, traditionally known for inter-strand crosslink repair, also regulates the Y-family polymerases. FANCD2 interacts with Pol η following UV and this is dependent on FANCD2 ubiquitination [135]. Pol η/FANCD2 interaction precedes Pol η/PCNA interaction following UV, suggesting FANCD2 is involved in the early regulation of Pol η. Recently, FANCD2 was shown to regulate chromatin localization of Pol η, but not Pol κ, following hydroxyurea [136]. REV1 also interacts with FANCD2, and loss of FANCD2 impairs REV1 accumulation at sites of laser microirradiation [137]. Interestingly, knock-down of *REV1* or *POLH* reduces FANCD2 foci following UV, consistent with co-regulation of FA proteins and Y-family polymerases. The FA core complex (A, B, C, E, G, F, M, and L) also regulates REV1 and Pol η. Following UV, FANCA and FANCG deficient cells display reduced REV1 foci compared to proficient cells [138]. Moreover, REV1 directly interacts with FAAP20, which stabilizes the FA core and promotes REV1 foci following UV. Additionally, the FA core may influence the expression of Pol η [139].

BRCA1 and BRCA2 have been extensively studied due to their roles in HR and dysregulation in breast cancers and are thought of as members of the FA pathway. In addition to lesion bypass, there is accumulating evidence that Y-family polymerases play an important role in the response to DSBs and are regulated in accordingly. BRCA1 was first reported to interact with REV1 and Pol η and its knock-down reduced their foci formation following UV [140]. BRCA2 as well as PALB2 were shown to interact with Pol η and co-localize following UV and replication stress, and to a lesser extent following IR [141]. Interaction with BRAC2/PALB2 was required for optimal Pol η foci formation following hydroxyurea. Both BRCA2 and PALB2, but not BRCA1, stimulated Pol η‘s DNA synthesis activity using a model recombination (D-loop) substrate in vitro.

## 6. Summary and Model for Orchestration

**Figure 3:**
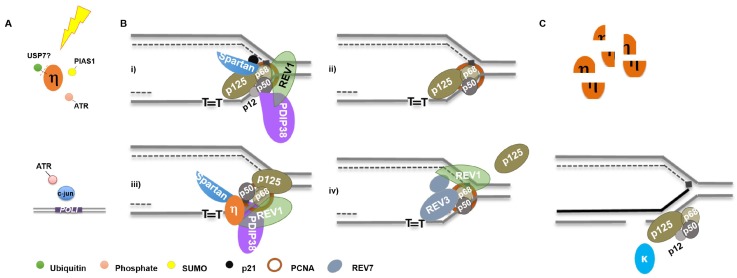
Orchestration of DNA Polymerases Following UV Irradiation: (**A**) following UV, Pol η is deubiquitinated, phosphorylated, and SUMOylated while *POLI* gene expression is induced; (**B**) thymine dimers stall replication forks requiring exchange, or altered polymerase activity (see text for details); and (**C**) following lesion bypass, Pol η is degraded by the proteasome while Pol κ and Pol δ (as well as Pol ε) filling in gaps generated by lesion incision by NER.

Our knowledge of how mammalian cells regulate the levels and activities of replicative and specialized polymerases to maintain genome integrity is in a state of infancy. Such regulation is quite intricate, and occurs at the transcriptional, post-transcriptional and translational levels (Figure 1). UV irradiation is the most well characterized model for polymerase orchestration (Figure 3). Following UV irradiation, Pol κ synthesis promotes ATR activation which, in turn, enhances Pol ι expression and phosphorylates Pol η [37,89,95]. This is likely concomitant with Pol η deubiquitination by USP7 and PIAS1/Rad18 dependent SUMOylation [108,111] (Figure 3A).

Recruitment of Pol η to sites of UV lesions is facilitated by numerous factors, as discussed above. REV1 may coordinate an exchange of DNA synthesis activity with Pol δ by interacting with p68 [125], with assistance by PDIP38/p50 and Spartan/p68 interactions (Figure 3Bi) [119,127,129,131]. Replicative Pol δ subunit composition is altered with the concomitant degradation of p12 and p21 (Figure 3Bii), which may aid polymerase orchestration at replication forks [85,142]. Pol η engagement at the fork may be facilitated by numerous protein-protein interactions (Figure 3Biii). Additionally, Pol ζ may be recruited to exchange subunits with Pol δ (p68 and p50) and assist in lesion bypass, in a manner likely facilitated by REV1 [143,144] (Figure 3Biv). Following lesion bypass, Pol η is degraded, while Pol κ and δ can participate in gap filling following lesion excision by NER (Figure 3C) [30,107]. The timing of PCNA mono-ubiquitination following UV irradiation and the nature of its function vis-à-vis polymerase orchestration is controversial [135,145,146,147]. Considering Pol η facilitates Rad18 recruitment to chromatin and it and Pol κ promote PCNA mono-ubiquitination, this modification may actually occur following specialized polymerase synthesis [60,120].

Much work remains to be done characterizing the orchestration of DNA polymerases during the replication stress response. However, research over the past decade clearly has shown this to be a more complex process than a single post-translational modification of PCNA. Numerous proteins converge on the Y-family polymerases to facilitate their recruitment to (presumably) stalled replication forks, including the Fanconi Anemia and BRCA pathways [136,141]. Interestingly, these pathways also promote repair of collapsed forks through DSBR and HR, and Y-family polymerases perform synthesis during these repair processes [31,36,148]. Therefore, recruitment of Y-family polymerases during replication stress may serve as an attempt to both relieve stalled replication and repair collapsed forks simultaneously. While such widespread utilization of error-prone polymerases may seem counter-intuitive, Pols η, κ, and ι interact with MMR proteins, opening the formal possibility that errors in the DNA synthesis products of these enzymes may be removed to maintain overall genome stability [32,36,149,150].

## Figures and Tables

**Table 1:** Known functions of mammalian replicative and specialized polymerases.

Polymerase	Gene/ Subunit	Cellular Functions	References
**Replicative**	**(B-family)**		
Alpha (α)	*POLA1-A2* p180, p70	Replication: initiator DNA synthesis	[23]
		Checkpoint signaling	[24,25]
Delta (δ)	*POLD1-D4* p125, p50, p66, p12	Replication: Lagging strand; late S/G2	[4,26]
		DNA repair synthesis (BER, NER, MMR)	[27,28,29]
		Checkpoint Signaling	[24]
Epsilon (ε)	*POLE1-E4* p261, p59, p17, p12	Replication: Leading strand	[4,26]
		DNA repair synthesis (NER)	[30]
		Checkpoint signaling	[24]
**Specialized**	**(B-family)**		
Zeta (ζ)	*REV3L*	Translesion synthesis Common Fragile Site stability	[1,13]
	*REV7*		
**Specialized**	**(Y-family)**		
Eta (η)	*POLH*	Translesion synthesis	[3]
		Common Fragile Site stability	[14]
		DNA repair synthesis (MMR; HR)	[31,32]
		Somatic Hypermutation	[33]
Kappa (κ)	*POLK*	Translesion synthesis	[3]
		G4 and Microsatellite DNA synthesis	[34]
		DNA repair synthesis (NER, DSBR)	[30,35,36]
		ATR signaling	[37]
Iota (ι)	*POLI*	Translesion synthesis	[3]
		Somatic Hypermutation	[38]
Rev1	*REV1*	Translesion synthesis	[3]
